# Biochemical Responses to the Long-Term Impact of Copper Sulfate (CuSO_4_) in Tobacco Plants

**DOI:** 10.3390/ijms242015129

**Published:** 2023-10-13

**Authors:** Anastasia S. Tugbaeva, Alexander A. Ermoshin, Irina S. Kiseleva

**Affiliations:** Department of Experimental Biology and Biotechnology, Institute of Natural Sciences and Mathematics, Ural Federal University, Ekaterinburg 620002, Russia; alexander.ermoshin@urfu.ru (A.A.E.); irina.kiseleva@urfu.ru (I.S.K.)

**Keywords:** copper stress, free and bound phenolics, secondary metabolites, antioxidant enzymes activity, lignification, *Nicotiana tabacum* L.

## Abstract

Metabolic changes under stress are often studied in short-term experiments, revealing rapid responses in gene expression, enzyme activity, and the amount of antioxidants. In a long-term experiment, it is possible to identify adaptive changes in both primary and secondary metabolism. In this study, we characterized the physiological state of tobacco plants and assessed the amount and spectrum of phenolic compounds and the lignification of axial organs under excess copper stress in a long-term experiment (40 days). Plants were treated with 100 and 300 μM CuSO_4_, as well as a control (Knop solution). Copper accumulation, the size and anatomical structure of organs, stress markers, and the activity of antioxidant enzymes were studied. Lignin content was determined with the cysteine-assisted sulfuric method (CASA), and the metabolite profile and phenolic spectrum were determined with UHPLC-MS and thin-layer chromatography (TLC). Cu^2+^ mainly accumulated in the roots and, to a lesser extent, in the shoots. Copper sulfate (100 μM) slightly stimulated stem and leaf growth. A higher concentration (300 μM) caused oxidative stress; H_2_O_2_ content, superoxide dismutase (SOD), and guaiacol peroxidase (GPOX) activity increased in roots, and malondialdehyde (MDA) increased in all organs. The deposition of lignin increased in the roots and stems compared with the control. The content of free phenolics, which could be used as substrates for lignification, declined. The proportions of ferulic, cinnamic, and *p*-coumaric acids in the hydrolysate of bound phenolics were higher, and they tended toward additional lignification. The metabolic profile changed in both roots and stems at both concentrations, and changed in leaves only at a concentration of 300 μM. Thus, changes in the phenolic spectrum and the enhanced lignification of cell walls in the metaxylem of axial (root and stem) organs in tobacco can be considered important metabolic responses to stress caused by excess CuSO_4_.

## 1. Introduction

The contamination of soil with heavy metals from industrial and agricultural activities and urbanization is a serious environmental problem [1,2,3,4]. As they accumulate in plants, metal ions are transmitted through food chains to animals and humans, adversely affecting their health [5]. Copper is a microelement and is necessary for the functioning of electron transport chains in mitochondria in all aerobic forms of life and in chloroplasts in photosynthetic organisms. Copper is a part of the reactive centers of many enzymes involved in various physiological processes [1,2,3,4]. When a certain concentration of this metal in plant tissues is exceeded, it causes a cytotoxic effect. Copper is a redox-active metal, and it catalyzes the formation of reactive oxygen species (ROSs) in the Fenton and Haber–Weiss reactions. Thus, an excess of copper can cause the development of an oxidative burst and subsequent stress. ROSs lead to the disruption of membrane integrity and transmembrane ion transport, imbalances in phytohormones, photosynthesis, and cellular respiration, the inhibition of plant growth and productivity, the browning of roots, and the chlorosis or necrosis of leaves [1,2,3,4]. Chronic exposure to excess copper over many generations can lead to heritable adaptations; shorter exposures cause acclimations. Plants limit the effects of stressors through various mechanisms, including genetic, epigenetic, biochemical, anatomical, morphological, and ontogenetic changes [6,7,8,9,10,11,12]. When a plant first encounters a stressor, it primarily responds by changing its metabolism.

Metabolic changes in plants under heavy metal stress are often studied in short-term laboratory experiments [6,7,8,9]. In such experiments, researchers evaluate stress indicators—the contents of hydrogen peroxide and malonaldehyde (as a product of the peroxidation of membrane phospholipids), enzyme activity, and the amounts of antioxidants. In a long-term experiment, it is possible to identify changes in metabolism for acclimation, including the compositions and amounts of different primary and secondary metabolites. In addition, plants can change the anatomy and morphology of their organs and physiological processes under stress [9,10,11,12,13]. 

Biochemical adaptations are also manifested with the rise in activity of antioxidant enzymes, such as SOD, catalase, ascorbate peroxidase, GPOX, etc., which leads to a decrease in the content of ROSs and the maintenance of redox balance in plant organs [8,9,14,15,16,17,18]. Plants’ responses to metal-induced stress may involve the synthesis of various primary and secondary metabolites. Plants modulate the formation of metabolites in response to environmental changes, which increases their viability and allows them to adapt to various stress factors. Thus, non-enzymatic antioxidants, including phenolic compounds, are involved in the neutralization of ROSs [6,7,15,16,17]. Phenolic compounds can chelate metals, which indirectly reduces the content of free radicals in plant cells. The activation of secondary metabolic pathways under the action of heavy metals, namely, the synthesis of phenylpropanoids, can lead to a change in the amount and spectrum of secondary phenolic metabolites, including lignin, one of the main components of plant cell walls, which, along with pectins, binds copper ions and limits their transport [10,12,15,19,20,21,22].

In conditions of excess heavy metals, including copper, the root acts as a barrier organ [9,10,15,16,20]. Plants bind and deposit copper ions in cell walls, translocate them to vacuoles, and allow them to accumulate there, thereby limiting long-distance transport from roots to shoots [2,18,20]. In response to the excess Cu^2+^ in the substrate, the anatomical structure of the roots and the architecture of the root system change [9,10,11,14,23]. Depending on the amount of copper in the substrate and the plant genotype, the density of lateral roots changes, which affects the surface area of the root system [9,10,11]. There is also an increase in lignification, which limits the growth of the roots in length and the translocation of ions to aboveground organs [10,11,13,15,16].

We assume that prolonged exposure to excess concentrations of CuSO_4_ can lead to a change in the qualitative and quantitative composition of phenolic compounds—for example, phenylpropanoid acids and derivatives of cinnamic acid are used as substrates for tissue lignification, which helps to limit the translocation of copper ions from roots to shoots and reduces the development of stress in leaves, which is important for maintaining the photosynthetic function in plants. In this regard, the aim of this study was to assess the amount and spectrum of phenolic compounds in plants under stress caused by an excess of copper in the environment via a long-term experiment (40 days), as well as to assess the lignification in the axial organs involved in the translocation of copper ions from the substrate to the leaves.

## 2. Results

The effects of the long-term interaction of copper sulfate with tobacco were first evaluated on a whole plant. The treatment of the substrate with 100 μM CuSO_4_ had a stimulating effect on the growth of tobacco, which led to significant increases in the total fresh weight by 13%, the plant height by 16%, and the leaf area by 13% compared to the control group of plants (Table 1 and Figure S1). On the contrary, at 300 μM CuSO_4_, the length of the main root decreased relative to the control, but lateral roots were intensively formed. The fresh weight of the plants, the stem height, and the leaf area did not change.

The general reaction of plants to excess CuSO_4_ in the medium was a thickening of the roots and stems. The effect was enhanced by an increase in the concentration of CuSO_4_ in the medium.

After the long-term CuSO_4_ treatment, the total copper content increased in the roots. In the cases of 100 and 300 µM, the total copper contents were 3.9 (111.25 ± 4.25 mg/kg) and 10 (281.89 ± 2.11 mg/kg) times higher compared to the untreated substrate (28.25 ± 0.75 mg/kg). The amount of available copper did not exceed 0.2% of its total content. It was shown [24,25] that at pH 5.3, copper ions bind hydroxyl groups of silicates and aluminosilicates in perlite and vermiculite.

In the tobacco plant, copper accumulated mainly in the root (Table 2). Its amount following the treatment with 100 and 300 μM CuSO_4_ increased by 23 and 26 times, respectively, in comparison with the control; in the stem, it increased by 2 and 3 times, and in the leaves by 2.8 and 3.5 times, respectively.

Copper was transported into the shoot in small amounts: the translocation factor (TF) for stems and leaves was less than 1, and the values of the bioconcentration factor (BCF) were also low (Table 2), which makes it possible to identify tobacco as a copper-excluder.

The amount of stress markers decreased under the treatment with 100 μM CuSO_4_ (Table 3). In the root, the H_2_O_2_ content declined by 29%, in the stem by 36%, and in leaves by 68% compared to the control. The marker of lipid peroxidation—MDA concentration—decreased by 16% in the roots compared to the control but did not change in the stems and leaves. On the contrary, under the treatment with 300 μM CuSO_4_, the amount of H_2_O_2_ increased in the root by 59%, and the amount of MDA increased by 18%. In the stem, hydrogen peroxide increased by 51%, and in the leaves, it increased by 109%, relative to the control.

**Figure 1:**
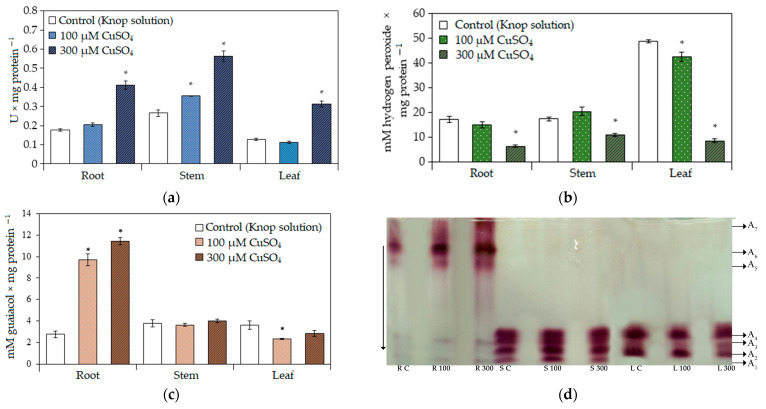
(**a**) The relative activity of superoxide dismutase, SOD. (**b**) The total activity of catalase. (**c**) The total activity of guaiacol peroxidases, GPOX. (**d**) The spectrum of isoforms and the total activity of class III peroxidases in tobacco plants after long-term substrate treatment with 100 and 300 μM CuSO_4_ solutions. The downward arrow indicates the direction of the electric current. Capital letters correspond to the organ type and treatment: R, root; S, stem; L, leaf; C, control; numbers correspond to CuSO_4_ concentrations (μM). The data in (**a**–**c**) are presented as the mean ± standard error, *n* = 5, and asterisks represent significant differences (*p* < 0.05, *U*-test) from control.

As the concentrations of stress markers were changed following the treatments, the activity of antioxidant enzymes was determined. At 100 μM CuSO_4_, the SOD activities in the roots and leaves were comparable to those in the control, and in the stem this activity increased by 33.6% (Figure 1a). At 300 μM CuSO_4_, the SOD activity increased in all organs: by 130.4% in the root, by 112.2% in the stem and by 142.0% in the leaves.

The total catalase activity was high in the leaf tissues in comparison with the axial organs (root and shoot) in all variants of the experiment (Figure 1b). At 100 μM CuSO_4_, the catalase activity in the roots and stems was comparable to that in the control, and in the leaf, it slightly decreased (Figure 1b). At 300 μM CuSO_4_, the catalase activity was decreased in all organs: by 63.3% in the roots, by 37.2% in the stems and 82.4% in the leaves. 

A different trend was found for the GPOX activity. The total activity of the enzyme increased (Figure 1c) in the roots under copper stress. In the shoot, it was approximately the same in the control and under the copper sulfate treatment.

Since the GPOX activity changed differently in tobacco roots and shoots, protein electrophoresis was performed for the visualization of peroxidase isoforms (Figure 1d). A_1_ and A_3_ were common in the leaves, stems, and roots (Figure 1d). Their activity increased in the roots under 100 and 300 µM CuSO_4_, but it decreased in the leaves treated with 300 µM CuSO_4_. The activity of the A_2_ isoform increased under stress in the leaves.

Three specific isoforms, A_5_, A_6_ and A_7_, were found in the root tissues. The activity of the A_5_ and A_6_ isoforms increased in all variants of the experiment compared to the control, and the A_7_ isoform was found only after the prolonged treatment of the substrate with 300 µM CuSO_4_. We suppose that the increase in the total GPOX activity in the roots was associated with the increase in the activity of the A_1_, A_3_, and A_5_ isoforms and the appearance of the A_7_ isoform under long-term treatment with 300 µM CuSO_4_.

In the stem, a unique A_4_ isoform was revealed, and it was characterized by high activity in all variants of the experiment. The activities of A_1_ and A_2_ isoforms increased at 100 µM CuSO_4_. A high level of GPOX isoform activity was found in both treated and control plants. 

Along with the changes in the physiological and biochemical characteristics of tobacco plants under copper stress, we found changes in the anatomy of the axial organs. Under the treatments with 100 and 300 μM CuSO_4_, the diameter of the stele increased by 73.2% and 65.5%, and the thickness of the cortex increased by 41.2–45.2%, in comparison with the control. As a result, the roots were thickened in the conduction zone (Table 4). The anatomy of the stem changed to a lesser extent. The thickness of the cortex in all variants of the experiment did not differ from the control. The stele diameter increased by 11.5 and 9.5%, and the xylem thickness increased by 39.0 and 38.6%, at 100 and 300 μM CuSO_4_, respectively.

**Figure 2:**
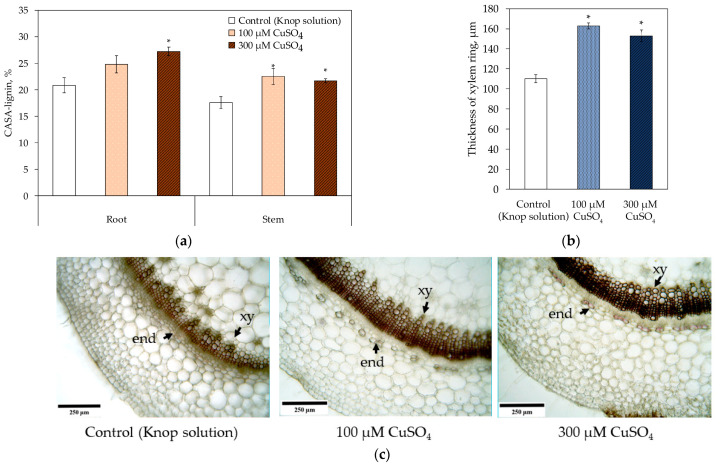
(**a**) CASA-lignin content in tobacco organs; the data are presented as the mean ± standard error, *n* = 5, and asterisks represent significant differences (*p* < 0.05, *U*-test) from the control. (**b**) The thickness of the xylem ring in the stems of tobacco plants; the data are presented as the mean ± standard error, *n* = 30, and asterisks represent significant differences (*p* < 0.05, *t*-test) from control. (**c**) The cross-sections of the stem. Lignin was stained with phloroglucinol hydrochloride. xy, xylem; end, endodermis. Bar 250 µm.

The prolonged action of copper stimulated the lignification of the tobacco roots and stems (Figure 2). In response to the copper treatment, the total content of CASA-lignin increased in the roots and stems compared with the control (Figure 2a). The lignification of the roots was more pronounced than that of the stems. Lignin was unevenly deposited in the cell walls of the endoderm. The thickness of the xylem ring, and the number of mechanical elements and xylem vessels in the vascular bundles, increased (Figure 2b,c).

The increased lignification of the root and stem tissues was accompanied by a decrease in the content of phenolic compounds (Table 5). Their content in the leaves was comparable to that in the control at 100 μM CuSO_4_, but significantly decreased at 300 μM CuSO_4_.

The qualitative composition of phenolic compounds (Table A1) shows the presence of benzoic and cinnamic acids, four derivatives of benzoic acid (gallic, vanillic, syringic and salicylic acids) and two derivatives of cinnamic acid (*p*-coumaric and ferulic acids).

Gallic, salicylic, vanillic acids, as well as ferulic and syringic acids were present in all plant organs under normal and stress conditions. Cinnamic acid was found in the roots of plants under the normal and stress conditions, and in the leaves it was found only under CuSO_4_ application. *p*-coumaric acid was also detected only in the experimental plants, and not in the control.

The analysis of the hydrolysate of cell walls showed that the content of the bound hydroxycinnamic acids in the roots decreased under the copper treatment in comparison with the control, which correlates with the increase in the lignification of cell walls. The ratios of cinnamic, *p*-coumaric and ferulic acids among the bound forms also increased. The contents of phenolic compounds in the hydrolysate were comparable to those in the control in the case of 100 μM CuSO_4_ in the stems and leaves. At 300 μM CuSO_4_, the ratios of ferulic and *p*-coumaric acids increased in the stems, but decreased in the leaves.

Since the tobacco organs differed in copper content, the metabolic profiles of the axial organs and the leaves have been analyzed (Table A2). Twenty individual compounds were identified. Under stress, the diversity of metabolites increased in all organs.

The leaves and the axial organs (root and stems) differed in the spectrum of metabolites. The highest metabolic similarity (coefficient 0.95) was found between the roots at 100 μM CuSO_4_ and 300 μM CuSO_4_ (Table 6). The metabolic similarity of the stems at these concentrations was also high, with a coefficient of 0.92. The metabolic profiles of leaves in the control and at 100 μM CuSO_4_ were also similar (coefficient 0.83).

**Figure 3:**
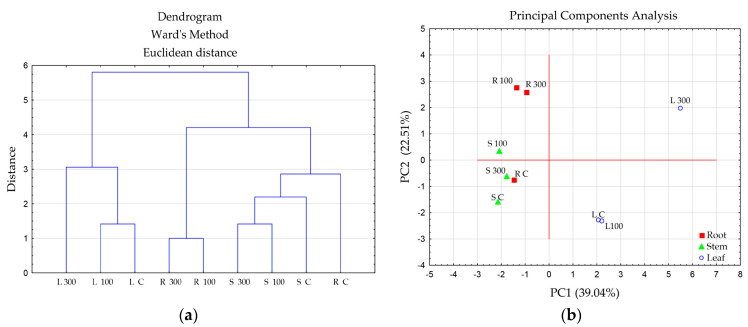
Multivariate data analysis based on UHPLC-MS analysis data of ethanol extracts from tobacco organs after the long-term substrate treatment with 100 and 300 μM CuSO_4_: (**a**) hierarchical clustering analysis; (**b**) score plot of principal component analysis using PC1 and PC2. Capital letters correspond to the organ type and treatment: R, root; S, stem; L, leaf; C, control; numbers correspond to CuSO_4_ concentrations (μM).

Figure 3 also demonstrates that the metabolic profile of the control differs from those of the treated plants, and the leaves are distinguished from the axial organs.

## 3. Discussion

Metabolic, anatomical, and morphological changes were recorded in tobacco under the prolonged treatment of plants with 100 and 300 μM CuSO_4_. These changes contributed to the plants’ adaptation to stress and survival during the 40-day period. Under these conditions, copper ions accumulated mainly in the roots, which indicates their barrier role. Thus, the transport of ions to the above-ground part of the plant was limited. Similar effects were seen both in the short-term and the long-term experiments for many copper-exclusive plants such as *Arabidopsis thaliana* L., *Brassica juncea* L., *Brassica napus* L., *Epipactis atrorubens* (Hoffm.) Besser., *Zinnia elegance* Jacq., etc. [9,10,13,16,17]. 

The effects of heavy metals on plants depend on the plant species, the genotype, the concentration of the metal and the duration of the stress factor [9,10,11,12,13,16,17]. In our study, the responses of tobacco plants to long-term exposure to 100 and 300 μM copper sulfate were different in the roots and in the shoots. Under low doses of copper sulfate (100 μM), plants successfully adapted. They did not differ from the control in terms of the total mass and length of the main root; moreover, the stem height and leaf area increased. We think that copper ions in low concentrations had a stimulating effect, since this element is essential for photosynthesis, respiration, and the absorption of macro- and microelements [9,11]. Under the low copper concentration, the stress markers, such as hydrogen peroxide and the level of lipid peroxidation (according to the amount of MDA), decreased in the roots in contrast to plants treated with 300 μM CuSO_4_. 

Under high doses of copper sulfate (300 μM), plants developed oxidative stress, as evidenced by the increase in hydrogen peroxide in the roots. In such plants, the levels of MDA and SOD activity in all organs were higher than in the untreated plants and in the case of 100 μM CuSO_4_. The rise in H_2_O_2_ concentration could be the result of the decreased catalase activity. The root system was more sensitive to excess copper in the medium (300 μM) compared with the stems and leaves, which corresponds to a significantly higher concentration of copper in this organ. Phenotypically, the effect of the stressor manifested in the browning of the root due to increased ROS production. At the same time, the leaves appeared undamaged—chlorosis and necrosis were not detected. Similar effects were described by other authors in *Trigonella foenum-graecum* [18], *B. juncea* and *B. napus* [9], and *Phyllostachys pubescens* (Pradelle) Mazel ex J.Houz. [26].

Excess copper caused structural changes in the anatomy of the root. The increases in the cortex and stele diameter and the thickness of the cell walls of xylem elements led to a thickening of the main root in the conduction zone. Since the stele consists mainly of xylem, its volume in the root also increased. These structural changes promoted the deposition of copper ions in the root and limited the long-distance transport of copper to the shoot. It is probable that the metal was deposed by binding with the hydroxyl and carboxyl groups of the cell wall components, as well as with amines, aldehydes, phosphates, and thiols [27,28]. Some of the absorbed copper ions were transported to the shoot. At 300 μM CuSO_4_, the copper concentration in the stem also became higher in comparison with the control. This led to changes in the anatomical characteristics of the stem: the diameter and stele volume, and the numbers of mechanical elements and vessels in the xylem, all increased.

Additional lignification was found in the axial organs of experimental plants. The deposition of lignin provided higher strength to the cell walls and an ability to bind copper ions, which protected the leaves from excesses of this metal. It is known that the lignification of cell walls is facilitated by the formation of hydrogen peroxide [9,21], and increases in the activity of class III peroxidases [29] and laccases [10,30]. Additional lignification under stress is also known in *A. thaliana*, *Glycine max* L., *Panax ginseng* C.A. Meyer, *Z. elegans*, etc. [10,13,31,32].

The synthesis of phenolic compounds plays an important role in plant resistance to stress, since they function as signal molecules and antioxidants, are precursors of lignin, and could chelate metal ions [6,12]. In tobacco, the contents of free phenolics in the root and stem decreased under excess copper, due to their participation in the enhanced lignification. The antioxidant activity of phenolic compounds depends on the number of hydroxyl groups in the molecule: cinnamic acid derivatives are more effective than benzoic acid derivatives [33]. In our experiments, *p*-coumaric acid was found in tobacco under stress conditions, and was not detected in the control. This could be a specific form of antioxidant under copper stress. Since phenolic compounds are involved in lignification, it is necessary to know the proportion of phenylpropanoids in the total amount of phenolics, as well as the amount and composition of bound phenolic compounds. In tobacco roots and shoots, the proportions of hydroxycinnamic acids among bound phenolics changed in response to prolonged exposure to copper sulfate. The increases in the amounts of ferulic, cinnamic and *p*-coumaric acids in root tissues in response to an excess of copper can be considered markers of the lignification process [34]. In our experiments, the rise of this proportion was more pronounced in the root than in the other tobacco organs, which enhanced its barrier function and the accumulation of copper [6].

Class III peroxidases play an important role in phenolic metabolism and cell wall lignification [29,31,32]. In tobacco roots and shoots, these enzymes differed in their isoform profile and their activity under copper stress compared to the control. A significant increase in the total activity of GPOX in root tissues contributed to the increased lignification of cell walls in this organ, and decreased the content of free phenolic compounds.

The biosynthesis of phenylpropanoid compounds depends on the expression of the *PAL*, *C4H*, *4CL*, *CCR*, and *CAD* gene families and the activity of the corresponding enzymes: phenylalanine ammonium lyase, cinnamate-4-hydroxylase, 4-coumarate-CoA ligase, cinnamoyl-CoA reductase, and cinnamoyl-alcohol dehydrogenase [35]. It was shown that excess copper differently impacted the expressions of these genes, or the activities of the enzymes in the roots or in the leaves, of *P. ginseng*, *Matricaria chamomilla* L., *Silene vulgaris* L., *Prosopis glandulosa* Torr., *Gynura procumbens* (Lour.) Merr., *Triticum aestivum* L., and *Z. elegans* [6,7,13,32,36,37,38]. In turn, these changes influenced the qualitative and quantitative composition of phenylpropanoid compounds. In our study, it was shown that the proportions of ferulic, cinnamic, and *p*-coumaric acids in the roots, stems, and leaves were different between the normal conditions and under stress.

The root and stem, being the axial organs, have similar anatomical structures, and provide for the transport of water, minerals and assimilates, while the leaf fundamentally differs from them in structure, functions and metabolism. Our data demonstrate that the composition of metabolites characterizes the specificity of organs. It was shown that the metabolic profiles of the root and stem in the control conditions are the most similar, and under stress, they were slightly different. In the leaves, the metabolic profiles of both the control and copper-impacted plants differed significantly from those in the axial organs.

The results obtained demonstrate that the biochemical, anatomical and morphological characteristics of the axial organs, associated with the acclimation to excess copper, had similar natures. The observed changes in the amounts and the compositions of phenolic compounds in the axial organs of tobacco, primarily the root, provided for the additional lignification of the cell walls, which increased the binding and deposition of copper ions and limited the xylem transport of ions from the root to the shoot.

## 4. Materials and Methods

### 4.1. Selection of Effective Concentrations and Growth Conditions

In a preliminary experiment, the seeds of *Nicotiana tabacum* L. cv. Petit Havana line SR1 were germinated in Petri dishes on filter paper discs with aqueous solutions 50, 100, 200, 300, 500, and 1000 μM CuSO_4_ with water as the control. On the 7th day the treatment of seeds with 50, 100 and 200 μM CuSO_4_ stimulated their germination by 23% to 8% relative to the control group of plants. At 300 μM CuSO_4_, seed germination did not change, and at 500 and 1000 μM CuSO_4_, it decreased significantly by 28 and 49%, respectively. Based on the results, the concentrations 100 and 300 μM CuSO_4_ were chosen for use in the long-term experiment to avoid acute toxicity during 40 days of tobacco cultivation.

In the long-term experiment, tobacco plants were cultivated in 0.2 L pots in the pre-autoclaved mixture of perlite and vermiculite (1:1) with Knop medium (control). The experimental plants were grown on Knop medium with the addition of aqueous solutions 100 and 300 μM CuSO_4_ for 40 days. The plants were irrigated every three days (30 mL solution). Tobacco plants were grown under the photoperiod 16 h (day):8 h (night), with temperature 23 ± 2 °C and humidity 65 ± 5%.

### 4.2. Anatomical and Morphological Characteristics of Plants

The stem height, root length, and area of the fifth leaf [39] were measured in 40-day-old plants. The fresh weight of the whole plant was determined with the gravimetric method; the dry weights of the root, stem and fifth leaf were determined after fixation at 110 °C and drying at 70 °C to constant weight. The number of biological replicates was at least 30 plants in each variant.

Fragments of the main root (mature zone) and stem (the fourth internode from the cotyledon leaves) at the age of 40 days were fixed in the mixture of 96% ethanol:glacial acetic acid (3:1, *v*/*v*) at 4 °C [40]. After 48 h, the samples were washed and stored in 96% ethanol at 4 °C and used for the anatomical analysis. Cross sections (100 µm-thick) were obtained with the freezing microtome MZP-01 (TECHNOM, Ekaterinburg, Russia). Lignin was stained with phloroglucinol–HCl [41]. The transverse sections of roots and stems were examined under a wide-field microscope Olympus BX51 WI (Olympus Corporation, Tokyo, Japan). Cell and tissue characteristics were studied using SIMAGIS^®^ Meso-Plant™ software version 2.1 for Windows XP. Five sections of each organ from one plant were analyzed. The total number of studied sections was at least 30 in each variant.

### 4.3. Quantification of Copper

The substrate was dried to a constant weight and then thoroughly ground and sieved (mesh diameter 2 mm). The total copper amount in the substrate was determined by digesting 0.25 g with HNO_3_:HClO_4_:HF (5:1:1, *v*/*v*/*v*) on a hot plate. The obtained solution was filtered through Whatman filter No. 42. The mobile forms of copper in the substrate were extracted by treating the samples with 4 mM Na_2_EDTA in a ratio of 1:25 (*w*/*v*) (shaken at 150 rpm for 24 h, pH 4.5). Extracts were acidified with 1% HNO_3_. To determine the copper concentration in tobacco organs (separately roots and stems), 50 mg of dried biomass (DW) was ashed in HNO_3_ [42]. 

The copper concentration in the solutions was determined with atomic emission spectroscopy (ICP-AES, iCAP 6500 Duo, Thermo Fisher, Waltham, MA, USA). The amount of copper ions in the substrate was expressed in mg copper kg^−1^, and in plant organs in µg g^−1^ DW. The BCF was determined in relative units as the ratio of Cu concentration in the organ (µg g^−1^ DW) to the amount of available Cu in the substrate (µg g^−1^). The TF was calculated as the ratio of Cu content in the stem or leaf (µg g^−1^ DW) to its concentration in the root (µg g^−1^ DW).

Pooled samples were taken from 3 plants for each variant of the treatment and the organ. The analysis of each sample was performed in 5 independent replicates.

### 4.4. Biochemical Characteristics

#### 4.4.1. Quantification of Hydrogen Peroxide and Malondialdehyde as Stress Markers

The concentration of hydrogen peroxide was determined via the oxidation of xylenol orange iron (III) chelates by peroxide, according to Bellincampi et al. [43], and expressed in µmol of hydrogen peroxide g^−1^ DW. The tissue extract for this test was prepared by rapidly stirring each sample in 0.1 M Tris-HCl buffer, pH 7.8.

The MDA concentration characterizes the intensity of lipid peroxidation. The amount of MDA was estimated spectrophotometrically in the reaction with thiobarbituric acid and expressed in µmol MDA g^−1^ DW [44]. 

#### 4.4.2. SOD, GPOX and Catalase Activity

The activity of the enzymes was assessed in crude enzyme extracts. The samples were frozen with liquid nitrogen and ground to a powder, and then immediately extracted with 100 mM phosphate buffer (pH 7.0). Then, the samples were centrifuged (12,000× *g*, 20 min, 4 °C); the supernatant was used for the assay of SOD, GPOX and catalase activity. 

SOD activity was measured spectrophotometrically using a method based on the inhibition of nitro tetrazolium blue photooxidation. The reaction medium (0.2 M phosphate buffer, pH 7.8) contained 39 μM L-methionine, 0.245 μM nitro tetrazolium, 0.3 μM EDTA and 0.025% Triton X-100. The activity was expressed in relative units as the rate of the reaction—rel. units/mg protein [45]. The optical density was measured at 560 nm.

The GPOX activity was assayed in the same crude extract in the reaction medium consisting of 0.1 M phosphate buffer (pH 7.0), 0.03% hydrogen peroxide and 0.1% guaiacol. Guaiacol was the hydrogen donor and hydrogen peroxide was the substrate. The optical density was measured at 470 nm. GPOX activity was expressed as mM guaiacol/mg protein × min [46]. 

Catalase activity was assayed using the same crude extract. The reaction medium (0.1 M phosphate buffer, pH 7.0) contained 0.03% hydrogen peroxide. The optical density was measured at 240 nm. Catalase activity was expressed as mM hydrogen peroxide/mg protein × min [46].

The amount of soluble protein was determined according to the Bradford method [47], using bovine serum albumin as a standard. All measurements of optical density were undertaken on a Shimadzu UV-1800 spectrophotometer (Shimadzu, Kyoto, Japan). All analyses were conducted in 3 biological (pooled samples from 5 plants) and 3 analytical replicates.

#### 4.4.3. Visualization of GPOX Isoforms 

The crude enzyme extracts were used for the non-denaturing 10% PAAG electrophoresis to reveal isoforms of III class peroxidases. For the staining, gels were placed in a reaction medium containing 0.2% benzidine or 0.2% guaiacol in 2% acetic acid [48]. To remove excess stains, the gels were washed in 2% acetic acid, then incubated for 3 min in 0.5% hydrogen peroxide until clear bands appeared against a non-stained background.

### 4.5. Determination of Total Lignin Content

The powdered plant material was treated with 96% ethanol for 5 h to remove extractives. The pellets were separated by centrifugation then dried to constant weight and used for lignin determination with the cysteine-assisted sulfuric method (CASA-lignin) [49]. Sulfuric acid (1.0 mL 72% H_2_SO_4_) with L-cysteine (0.1 g mL^−1^) was added to 10 ± 0.1 mg of plant material. The mixture was incubated and constantly stirred at 24 °C until complete dissolution of the plant material (60–70 min). The resulting solution was transferred to a volumetric flask and diluted with deionized water to 100 mL. The optical density of solutions was measured at 283 nm on a Shimadzu UV-1800 spectrophotometer (Shimadzu, Kyoto, Japan). The calculation of the lignin content was done using the extinction coefficients 11.26 (guaiacyl units: syringyl units ratios ≤ 1) [50,51]. The analysis was performed in 3 biological (pooled samples from 15 plants) and 3 analytical replicates.

### 4.6. Phenolic Compounds

Free phenolic compounds were extracted from the dry plant material with ethanol and a biomass/solvent ratio 1:10. The extraction time was 30 min, and the was temperature 55 °C. Extraction was performed three times, and the samples were pooled. The contents of the total phenolic compounds were assayed spectrophotometrically in the reaction with the Folin–Ciocalteu reagent [52], modified for the microplate reader [29].

The qualitative composition of phenolic compounds was determined with the UHPLC-MS method (Xevo QTof MS, Waters, Milford, MA, USA). Chromatographic separation was carried out in an acetonitrile–1% formic acid gradient on a C-8 reverse-phase column. The extracts were passed through a C-18 reverse phase pre-column and a microfilter with 0.22 μM pore size prior to analysis.

Cinnamic acid esters and glycosides bound to cell walls were identified according to a modified method [53], after the saponification of plant material with 5% sodium hydroxide ethanol solution and hydrolysis of the residue with the concentrated hydrochloric acid. The fractions were pooled, washed with distilled water, and the pH was adjusted to neutral. The hydrolysate was then re-extracted three times into ethyl acetate, dried, and dissolved in a small volume of methanol [29]. The hydrolysate was separated using silica gel TLC in an ethyl acetate–toluene–formic acid solvent system. The zones corresponding to the standards of ferulic, *p*-coumaric and cinnamic acids were eluted into methanol, and their amount was determined spectrophotometrically on a Shimadzu UV-1800 spectrophotometer (Shimadzu, Kyoto, Japan). The ratio of hydroxycinnamic acids to the total amount of phenolic compounds in the ethyl acetate fraction in the hydrolysate [29] was calculated.

### 4.7. Metabolic Fingerprinting 

HPLC-MS chromatograms were used not only for the qualitative analysis of phenolic compounds, but also for the comparison of the metabolic profiles of samples. For this, the total ion current (TIC-) chromatograms based on the GC-MS data were analyzed. A binary matrix was built according to the molecular weight of the major metabolites; the coefficient of similarity was calculated and it was used for constructing a dendrogram [54].

### 4.8. Statistical Analysis

The experiment was repeated three times. The data are presented as the arithmetic mean values and the standard error. Statistical data processing was carried out in STATISTICA 13 (StatSoft Incorporated, Tulsa, OK, USA) using Student’s *t*-test and Mann–Whitney *U*-test, with asterisks indicating significant differences from the control (*p* < 0.05). The data of the metabolic fingerprinting were analyzed by hierarchical clustering analysis and principal component analysis. Figures were prepared using Microsoft Excel 2021 (Microsoft Corporation, Redmond, WA, USA), STATISTICA 13 (StatSoft Incorporated, Tulsa, OK, USA) and Adobe Photoshop 2020 (Adobe Systems Incorporated, San Jose, CA, USA). 

## 5. Conclusions

One of the non-specific reactions of excluder plants to heavy metals stress is the strengthening of the root barrier function by the accumulation of the metals in root tissues. Heavy metals can be bound in vacuoles, cytoplasm, and cell walls. Our study showed that under copper stress, metabolic changes occurred in the axial organs of plants (roots, stems). These changes were associated with the composition and amount of phenolic compounds and lignin. The proportions of ferulic, cinnamic, and *p*-coumaric acids, which are the precursors of lignin biosynthesis, increased under excess copper treatment. These changes enhanced the barrier function of the root and limited the transport of copper ions to the leaves. Moreover, such metabolic changes modified the structural characteristics of the tissues (thickening of the root, stem, cell walls of the metaxylem vessels due to the deposition of CASA lignin, etc.). The number of individual metabolites in plant organs increased under prolonged copper stress. Similarities between the metabolic profiles in the roots and stems were found both under control conditions and under excess CuSO_4_.

Thus, in tobacco, the biochemical responses to copper stress included specific changes in the spectrum of phenolic compounds and the amount of lignin in the axial organs.

## Figures and Tables

**Table 1:** Morphological parameters of tobacco plants after the long-term substrate treatment with 100 and 300 μM CuSO_4_.

Treatment	Root Length, cm	Root Diameter, μm	Stem Height, cm	Stem Diameter, μm	Area of the 5th Leaf, cm^2^
Control (Knop solution)	10.5 ± 0.5 ^1^	1146 ± 58	11.1 ± 0.4	2598 ± 13	61.0 ± 2.2
100 μM CuSO_4_	9.6 ± 0.8	1593 ± 45 *	12.9 ± 0.5 *	2609 ± 29 *	69.0 ± 2.4 *
300 μM CuSO_4_	7.3 ± 0.8 *	1764 ± 64 *	11.6 ± 0.6	2787 ± 24 *	57.0 ± 2.3

^1^ Results are presented as mean ± standard error (*n* = 30). * represent significant differences (*p* < 0.05, *t*-test) from control.

**Table 2:** Amount of copper in tobacco organs; BCF and TF after the long-term substrate treatment with 100 and 300 μM CuSO_4_.

Treatment	Copper Amount, µg g^−1^ DW			BCF			TF	
	Root	Stem	Leaf	Root	Stem	Leaf	Stem/Root	Leaf/Root
Control (Knop solution)	31.36 ± 1.09 ^1^	14.84 ± 0.56	9.52 ± 0.44	1.11	0.53	0.34	0.47	0.30
100 μM CuSO_4_	723.24 ± 39.78 *	31.08 ± 1.31 *	27.44 ± 1.26 *	2.28	0.10	0.09	0.04	0.04
300 μM CuSO_4_	810.88 ± 32.43 *	44.80 ± 2.46 *	33.21 ± 1.86 *	1.01	0.06	0.04	0.06	0.04

^1^ Results are presented as mean ± standard error (*n* = 5). * represent significant differences (*p* < 0.05, *U*-test) from control.

**Table 3:** The amount of hydrogen peroxide and MDA in tobacco organs after the long-term substrate treatment with 100 and 300 μM CuSO_4_.

Treatment	H_2_O_2_, µmol g^−1^ DW			MDA, µmol g^−1^ DW		
	Root	Stem	Leaf	Root	Stem	Leaf
Control (Knop solution)	7.70 ± 0.19 ^1^	8.51 ± 0.29	37.23 ± 0.76	42.40 ± 1.59	22.28 ± 1.01	28.74 ± 2.31
100 μM CuSO_4_	5.43 ± 0.16 *	5.45 ± 0.28 *	12.05 ± 0.16 *	35.46 ± 1.61 *	23.83 ± 0.61	25.79 ± 1.50
300 μM CuSO_4_	12.28 ± 0.29 *	7.55 ± 0.14	25.24 ± 0.55 *	49.90 ± 0.55 *	33.63 ± 1.74 *	59.99 ± 2.68 *

^1^ Results are presented as mean ± standard error (*n* = 5). * represent significant differences (*p* < 0.05, *U*-test) from control.

**Table 4:** Anatomy characteristics of tobacco organs after the long-term substrate treatment with 100 and 300 μM CuSO_4_.

Treatment	Root				Stem			
	Stele Cross-Sectional Diameter, mm	Stele Area, %	Cortex Thickness, µm	Cell Wall Thickness of Metaxylem Vessels, µm	Stele Cross-Sectional Diameter, mm	Stele Area, %	Cortex Thickness, µm	Cell Wall Thickness of Metaxylem Vessels, µm
Control (Knop solution)	650 ± 42 ^1^	32.2	172 ± 8	2.74 ± 0.03	1738 ± 19	44.7	403 ± 18	2.91 ± 0.14
100 μM CuSO_4_	1326 ± 47 *	38.5	250 ± 13 *	2.94 ± 0.10 *	1938 ± 20 *	48.0	395 ± 11	2.97 ± 0.06
300 μM CuSO_4_	1077 ± 41 *	37.3	244 ± 10 *	2.92 ± 0.09 *	1903 ± 41 *	46.6	415 ± 19	3.21 ± 0.08 *

^1^ Results are presented as mean ± standard error (*n* = 30). * represent significant differences (*p* < 0.05, *t*-test) from control.

**Table 5:** The contents of free and bound phenolics (mg g^−1^ DW) in tobacco organs after the long-term substrate treatment with 100 and 300 μM CuSO_4_ solutions.

Organ	Treatment	Free Phenolics	Bound Phenolics	Proportions (%) of Phenolic Acids in Hydrolysate		
				Ferulic Acid	Cinnamic Acid	*p*-Coumaric Acid
Root	Control (Knop solution)	62.35 ± 2.66 ^1^	7.13 ± 0.18	5.6	2.5	4.5
	100 μM CuSO_4_	49.30 ± 1.07 *	4.42 ± 0.05 *	9.8	8.2	10.8
	300 μM CuSO_4_	56.39 ± 2.11 *	4.96 ± 0.05 *	7.0	5.2	9.7
Stem	Control (Knop solution)	65.65 ± 1.77	3.44 ± 0.08	5.7	6.7	9.7
	100 μM CuSO_4_	36.60 ± 1.04 *	3.31 ± 0.03	7.6	3.5	7.4
	300 μM CuSO_4_	44.23 ± 2.02 *	4.78 ± 0.05 *	3.2	1.8	2.8
Leaf	Control (Knop solution)	37.10 ± 2.13	8.39 ± 0.12	39.5	21.8	25.3
	100 μM CuSO_4_	36.01 ± 2.73	7.57 ± 0.23	31.4	12.6	28.3
	300 μM CuSO_4_	30.17 ± 0.91 *	4.74 ± 0.13 *	7.0	6.1	6.9

^1^ Results are presented as mean ± standard error (*n* = 5 for free phenolics, *n* = 3 for bound phenolics). * represent significant differences (*p* < 0.05, *U*-test) from control.

**Table 6:** Metabolic similarity coefficient of tobacco organs after the long-term substrate treatment with 100 and 300 μM CuSO_4_.

Name		Root			Stem			Leaf		
		Control (Knop s.)	100 μM CuSO_4_	300 μM CuSO_4_	Control (Knop s.)	100 μM CuSO_4_	300 μM CuSO_4_	Control (Knop s.)	100 μM CuSO_4_	300 μM CuSO_4_
Root	Control (Knop s.)	1	0.67	0.59	0.67	0.63	0.67	0.43	0.33	0.34
	100 μM CuSO_4_		1	0.95	0.53	0.78	0.70	0.44	0.38	0.48
	300 μM CuSO_4_			1	0.56	0.82	0.64	0.47	0.40	0.50
Stem	Control (Knop s.)				1	0.80	0.80	0.40	0.31	0.22
	100 μM CuSO_4_					1	0.92	0.53	0.35	0.45
	300 μM CuSO_4_						1	0.53	0.47	0.45
Leaf	Control (Knop s.)							1	0.83	0.59
	100 μM CuSO_4_								1	0.53
	300 μM CuSO_4_									1

## Data Availability

Not applicable.

## Supplementary Materials

The following supporting information can be downloaded at: https://www.mdpi.com/article/10.3390/ijms242015129/s1.

## Appendix A

Spectrum of metabolites of tobacco plant organs after long-term substrate treatment with 100 and 300 μM CuSO_4_ solutions. Table A1 shows phenolic compounds identified by UHPLC-MS method. Table A2 shows analytes detected by UHPLC-MS method.

**Table A1:** The binary matrix of phenolic compounds identified by UHPLC-MS (1—present, 0—absent).

Compound					Root			Stem			Leaf		
№	Rt, min	m/z, [M-H]-	Formula	Name	Control	Cu 100 µM	Cu 300 µM	Control	Cu 100 µM	Cu 300 µM	Control	Cu 100 µM	Cu 300 µM
1	2.09	289.07	C_15_H_14_O_6_	Catechin	1	0	0	0	0	0	0	0	0
2	10.05	301.03	C_15_H_10_O_7_	Quercetin	0	0	0	0	0	0	0	0	0
3	5.92	609.15	C_27_H_30_O_16_	Rutin	1	1	1	1	1	1	1	1	1
4	8.3	227.07	C_14_H_12_O_3_	Resveratrol	1	0	0	0	0	0	0	1	1
5	0.64	169.01	C_7_H_6_O_5_	Gallic acid	1	1	1	1	1	1	1	1	1
6	5.6	137.02	C_7_H_6_O_3_	Salicylic acid	1	1	1	1	1	1	1	1	1
7	9.23	147.04	C_9_H_8_O_2_	Cinnamic acid	1	1	1	1	1	1	1	1	1
8	3.96	163.04	C_9_H_8_O_3_	*p*-Coumaric acid	1	1	1	0	0	0	0	1	1
9	4.61	193.05	C_10_H_10_O_4_	Ferulic acid	0	1	1	0	1	1	0	1	1
10	2.34	167.03	C_8_H_8_O_4_	Vanillic acid	1	1	1	1	1	1	1	1	1
11	2.74	197.05	C_9_H_10_O_5_	Syringic acid	1	1	1	1	1	1	1	1	1
12	4.91	121.01	C_7_H_6_O_2_	Benzoic acid	1	1	1	1	1	1	1	1	1
13	0.62	125.02	C_6_H_6_O_3_	Phloroglucinol	1	1	1	1	1	1	1	1	1

**Table A2:** The binary matrix of analytes detected by UHPLC-MS (1—present, 0—absent).

Compounds			Root			Stem			Leaf		
№	Rt, min	m/z, [M-H]-	Control	Cu 100 µM	Cu 300 µM	Control	Cu 100 µM	Cu 300 µM	Control	Cu 100 µM	Cu 300 µM
1	0.61	341.11	0	1	1	0	1	1	1	1	1
3	0.87	249.13	1	1	1	1	1	1	0	0	1
3	1.08	146.96	0	0	0	0	0	0	0	0	1
4	1.3	203.09	0	0	0	0	0	1	1	1	1
5	2.07	191.04	1	1	1	1	1	1	1	1	1
6	2.52	121.03	0	1	1	0	0	0	0	0	0
7	2.82	472.15	0	0	0	1	1	1	0	0	0
8	2.91	401.15	0	1	1	0	1	0	1	0	1
9	3.16	194.91	1	1	1	0	0	0	0	0	0
10	3.48	470.24	1	0	0	1	1	1	1	0	0
11	3.81	468.22	0	0	0	0	0	0	0	0	1
12	3.89	468.22	0	0	0	1	1	1	0	0	0
13	4.22	176.02	0	1	1	0	1	1	0	0	1
14	7.38	187.11	0	0	0	0	0	0	1	1	0
15	7.51	187.09	1	1	1	1	1	1	1	1	1
16	8.78	282.12	0	1	1	1	1	1	0	0	0
17	9.33	312.13	1	1	1	1	1	1	0	0	0
18	9.78	301.04	1	1	0	0	1	1	0	0	0
