# Supplementary material for: Biochemical Responses to the Long-Term Impact of Copper Sulfate (CuSO4) in Tobacco Plants
Source: Int J Mol Sci. 2023 Oct 13;24(20):15129. doi: 10.3390/ijms242015129 (PMC10606774; doi:10.3390/ijms242015129)
Supplement: Supplementary file 1 [file ijms-24-15129-s001.zip › ijms-2628690-supplementary.pdf]

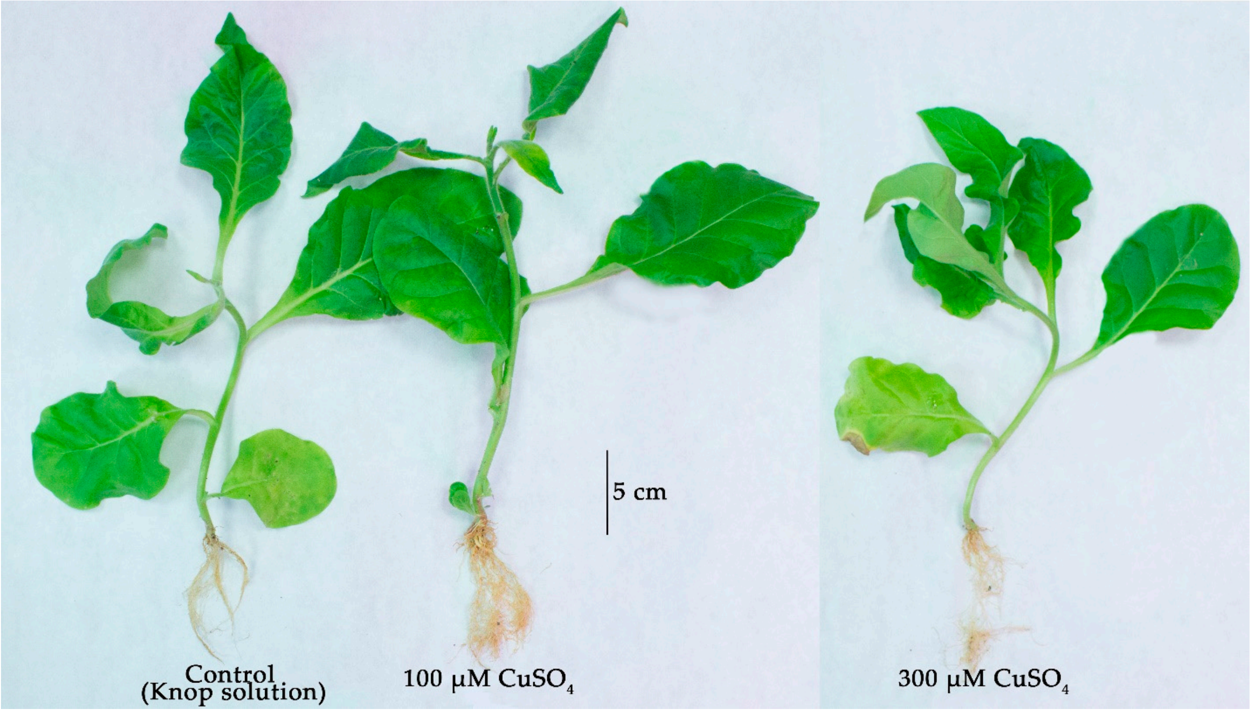

Figure S1. Tobacco plants after the long-term substrate treatment with 100 and 300 μM $CuSO_4$. Bar 5 cm.
